# The Usefulness of Outpatient Cardiac Telemetry in Patients with Cryptogenic Stroke

**DOI:** 10.3390/jcm13133819

**Published:** 2024-06-28

**Authors:** Anetta Lasek-Bal, Adam Konka, Przemysław Puz, Joanna Boidol, Katarzyna Kosarz-Lanczek, Agnieszka Puz, Anna Wagner-Kusz, Andrzej Tomasik, Sebastian Student

**Affiliations:** 1 Department of Neurology, School of Health Sciences, Medical University of Silesia in Katowice, 40-055 Katowice, Poland; 2 Upper-Silesian Medical Centre of the Silesian Medical University in Katowice, 40-055 Katowice, Poland; doma.l@poczta.onet.pl (K.K.-L.);; 3 Silesian Park of Medical Technology Kardio-Med Silesia, 42-800 Zabrze, Poland; a.konka@kmptm.pl (A.K.); tomasik@poczta.onet.pl (A.T.); 4 1st Department of Cardiology and Angiology, Silesian Center for Heart Diseases, 41-800 Zabrze, Poland; 5 2nd Department of Cardiology in Zabrze, Faculty of Medical Sciences in Zabrze, Medical University of Silesia in Katowice, 40-055 Katowice, Poland; 6 Faculty of Automatic Control, Electronics and Computer Science, Silesian University of Technology, 44-100 Gliwice, Poland; sebastian.student@polsl.pl; 7 Biotechnology Center, Silesian University of Technology, 44-100 Gliwice, Poland

**Keywords:** atrial fibrillation, cryptogenic stroke, embolic stroke, ESUS

## Abstract

**Introduction**: Atrial fibrillation (AF), apart from non-stenotic supracardiac atherosclerosis and neoplastic disease, is the leading cause of cryptogenic stroke, including embolic stroke of un-determined source (ESUS). The aim of our study was to determine the prevalence of AF in ESUS patients based on 30-day telemetric heart rate monitoring initiated within three months after stroke onset. Another aim was to identify factors that increase the likelihood of detecting subsequent AF among ESUS patients. **Material and Methods**: patients with first-ever stroke classified as per the ESUS definition were eligible for this study. All patients underwent outpatient 30-day telemetric heart rate monitoring. **Results**: In the period between 2020 and 2022, 145 patients were included. The mean age of all qualified patients was 54; 40% of eligible patients were female. Six patients (4.14%), mostly male patients (4 vs. 2), were diagnosed with AF within the study period. In each case, the diagnosis related to a patient whose stroke occurred in the course of large vessel occlusion. Episodes of AF were detected between day 1 and 25 after starting ECG monitoring. Out of the analyzed parameters that increase the probability of, A.F.; only supraventricular extrasystoles proved to be an independent factor regarding an increased risk of AF [OR 1.046, CI 95% 1.016–1.071, *p*-value < 0.01]. **Conclusions**: The use of telemetry heart rhythm monitoring in an outpatient setting can detect AF in 4% of ESUS patients who have undergone prior diagnostic procedures for cardiogenic embolism. Supraventricular extrasystoles significantly increases the likelihood of AF detection in patients with ESUS within three months following stroke. Comorbid coronary artery disease, diabetes and hypertension, rather than a single-factor clinical burden, increase the likelihood of AF detection in older ESUS patients. ESUS in the course of large vessel occlusion is probably associated with an increased likelihood of cardiogenic embolism.

## 1. Introduction

When planning rational and effective secondary prevention, it is fundamental to determine the etiology of stroke. However, despite appropriate diagnostics including cardiovascular imaging and 24 to 48 h Holter cardiac monitoring, the cause of stroke cannot be determined in about 25% of patients (cryptogenic stroke). Embolic stroke of undetermined source (ESUS) is a type of cryptogenic stroke resulting from an embolic mechanism similar to that in cardiogenic stroke [1]. Clinical trials focused on the efficacy of anticoagulation in patients with cryptogenic stroke necessitated the identification of ESUS among a heterogeneous group of cryptogenic strokes. It was expected that a high percentage of ESUS patients have covert atrial fibrillation; therefore, anticoagulation as secondary prevention is needed.

Stroke is related to atrial fibrillation (AF) in 18–23% of all ischemic stroke patients [2,3,4]. That group includes patients with AF identified before stroke onset or during causative diagnostics in hospitalized patients or subsequent outpatient care. It has been proven that even short (lasting several minutes) asymptomatic episodes of AF increase the risk of stroke by 2.8 times and the risk of death by 2.5 times [5]. Due to the clinical relevance of, A.F.; an international group of experts, AF-SCREEN, was formed in 2015. The group defined strategies for identifying AF [6]. 

The following methods are used to detect AF: palpated pulse measurement, the standard 12-lead electrocardiogram (ECG), a 24 h Holter ECG, a Holter ECG for several days, an event monitor (on-demand monitoring function or auto-detect feature), an implantable loop recorder (ILR) and implanted electrotherapy devices to detect atrial electrical activity (i.e., pacemaker, implantable cardioverter-defibrillator [ICD], cardiac resynchronization therapy [CRT] pacemaker).

Numerous technologies are readily accessible to detect heart rhythm disturbances.

The currently available devices for detecting atrial fibrillation can be divided into three categories: (1) long-term (>14 days) non-invasive ECG monitors; (2) cardiac rhythm monitoring devices equipped with software that runs algorithms to detect AF; and (3) technologies other than ECG.

Category 1 includes the PocketECG system by Medicalgorithmics and the Nomed-AF system developed in Poland by the consortium of Silesian Park of Medical Technology Kardio-Med Silesia, Medical University of Gdansk, Medical University of Warsaw, Jagiellonian University Medical College, Pomeranian Medical University, Institute of Medical Technology and Equipment, and Comarch Healthcare. The Nomed-AF project (“Non-invasive Monitoring for Early Detection of Atrial Fibrillation)”–ClinicalTrials.gov number NCT03243474 provides solutions for long-term 30-day monitoring of electrocardiogram signals.

Category 2 includes the Apple Watch Series 4 with AliveCor KardiaBand wristband and Sky Labs CART Ring. Category 3 includes medical apps that provide heart rhythm monitoring using a smartphone camera (FibriCheck (https://www.fibricheck.com/), Heartbeats software (https://heartbeat.software/), etc.).

These devices have varying sensitivity and specificity in detecting arrhythmias—which can lead to false-positive results and unnecessary treatment [7].

Diagnosing AF is crucial for primary and secondary stroke prevention, as oral anti-coagulants are highly effective in preventing strokes.

As demonstrated, ischemic stroke due to AF is more severe than strokes of other eti-ologies. Since ESUS has an embolic nature (proximal clot), the diagnostic procedures for AF should be intensified in patients with ESUS. Considering how difficult it is to detect short-term asymptomatic and paroxysmal AF using traditional monitoring, various pro-longed heart rate monitoring formulas are used. Determining the cause of cerebral ischemia in ESUS patients is important due to the choice of secondary prevention which should be strictly based on the cause of a prior stroke. Today, we already know that the incidence of AF among ESUS patients is lower than expected. The results of the NAVIGATE ESUS and RESPECT ESUS studies confirm that the benefits of anticoagulation in a group of selected patients are no greater than those of aspirin use [8,9]. It is possible that patients with ESUS and a cardiogenic thromboembolic risk profile may benefit from anticoagulants. However, this is not supported by the preliminary results of the ATTICUS study [10]. We are eagerly awaiting the results of the ARCADIA and MOSES studies, which are expected to shed light on the potential benefits of anticoagulation in post-ESUS patients with atrial cardiopathy without AF but at a high risk of left-atrial thromboembolism [11].

Before the definition of ESUS can be revised and an algorithm for post-ESUS management established based on randomized trials using modern instrument technologies, AF detection is strongly advisable.

The aim of our study was to determine the prevalence of AF in ESUS patients based on 30-day telemetric heart rate monitoring initiated within three months after stroke onset. Another aim was to identify factors that increase the likelihood of detecting subsequent AF among ESUS patients.

## 2. Material and Methods

Patients with first-ever ischemic stroke classified as cryptogenic as per the ESUS definition were eligible for this study [1,2]. The diagnosis of stroke was confirmed based on clinical presentation and imaging findings (head CT and/or MRI). In addition to these tests, all patients undergoing the diagnosis of stroke cause (performed ≤ 3 months prior to study inclusion) underwent the following procedures: CT angiography of the aortic arch, carotid and cerebral arteries; transthoracic and transesophageal echocardiography; ultra-sound of the carotid and vertebral arteries; ECG; 24 and 48 h Holter monitor; basic blood count tests; tests for coagulation disorders, connective tissue diseases and infections; and basic imaging and neoplastic tumor markers. Eligibility was based on the following: informed consent to participate in the study; no previously detected atrial fibrillation/flutter (prior to stroke and during hospitalization at least, based on 48 h Holter ECG recording); and stroke onset within no more than 3 months after study inclusion. Patients who had experienced a transient ischemic attack (TIA) or who required permanent anti-coagulation for reasons other than AF were not eligible. For the purposes of this study, it was established that a patent foramen ovale was a disqualifying factor. During the eligibility period, all patients used antiplatelet therapy (monotherapy or dual therapy) as a secondary prevention of stroke; they also used statins.

The patients who qualified underwent 30-day telemetric heart rate monitoring and had a neurological and cardiological consultation. They also consulted a rehabilitation specialist twice—before starting remote cardiac monitoring (0–3 days) and during the end-of-study visit (day 35 ± 5).

### 2.1. Description of the Cardiac Monitoring Procedure

Bittium’s Cardiac Navigator system (Faros 360°) was used in Holter ECG analysis for long-term (30-day) ECG monitoring.

Each patient received a telemonitoring kit consisting of the following: two Faros sensors, with the software and additional accessories (set of 5-electrode cables for 3-channel ECG, two chargers); and a receiving station (smartphone).

Patients and their families were shown how to use the technology.

All participants were encouraged to wear the system for as long as they could, with short breaks for necessary reasons, such as daily hygiene practices.

ECG recordings every 30 min were transmitted to a smartphone and then transferred online to a telemonitoring center of the Mediguard telemedicine platform. The data trans-mitted were analyzed and presented to the specialists evaluating ECG recordings.

Each of the detected episodes was reviewed and finally confirmed as AF by cardiologists.

According to current ESC guidelines, only subjects with AF episodes lasting at least 30 s were included in the analysis as AF-positive individuals [12,13]. Silent AF was defined as AF that was detected and confirmed by expert cardiologists in asymptomatic participants.

The diagnosis of AF served as an endpoint in the study and as a reason for initiating oral anticoagulant therapy.

### 2.2. Description of Statistical Methods

Logistic regression analysis was performed to identify any potential independent risk factors for AF among the following: age, sex, nicotinism, coronary artery disease, peripheral artery disease, diabetes mellitus, arterial hypertension, lipid disorders, past myocardial infarction, post-stroke functional status as per the modified Rankin Scale (mRS), NIHSS on day 1 of stroke and the day of study inclusion, stroke location, stroke type, peripheral atherosclerosis, sinus bradycardia, premature atrial complexes, excessive supra-ventricular electric activity, increased maximum P-wave duration, prolonged PR interval and interatrial conduction block.

Multivariable models were built by using binary logistic regression for binary out-comes. The model variable selection procedures included automatic selection (stepwise, forward and backward) based on the AIC (Akaike information criterion) and BIC (Bayesian information criterion) [14,15]. For the evaluation of the accuracy of model predictions, a “leave-one-out” procedure to avoid data leakage so as not to cause over-fitting and the AUC (area under the ROC curve) estimator were used [16,17]. All statistical analyses were performed using R version 3.6.1.

The study was approved by the Committee for Bioethics in Medicine at the Silesian Medical Chamber in Katowice—Resolution No. 39/2020, dated 27 October 2020.

## 3. Results

Patients were recruited among those treated at the Center for Interventional Stroke Treatment, Upper-Silesian Medical Center (GCM) in Katowice. In the period between 2020 and 2022, 150 patients were initially included. However, 145 patients completed the study (five patients resigned), so the data from 145 patients were included in the analyses planned for the study. In that period, 1286 ischemic stroke patients were treated at GCM; 201 patients (15.62%) were diagnosed with AF before stroke onset and 116 patients (9.02%) were diagnosed with AF based on diagnostic procedures performed during hospitalization.

Patients enrolled in the presented study accounted for 11.6% of all patients treated for ischemic stroke at the study site within the study period. Forty percent of eligible patients were female. The mean age of all qualified patients was 54 (SD = 12.91).

The study patients are described in Table 1.

Six patients (4.14%) were diagnosed with AF within the study period. In each case, the diagnosis related to a patient whose stroke occurred in the course of large vessel occlusion.

The parameters for telemetry patient monitoring are presented in Table 2.

In 67% of patients, AF was diagnosed between 7 and 25 days after the start of recording, with an average diagnosis made on day 11. The diagnosis of AF was made between nine and 39 days after the first symptoms of stroke, with an average diagnosis on day 13. The time of first AF episode was ranged from 23 min to 24 h.

The mean age of the study population in whom AF was detected was 62 (SD = 11.51) years and it comprised mostly male patients (4 vs. 2); 83% of patients diagnosed with AF had a history of arterial hypertension. In patients with AF detected, oral anticoagulant therapy as well as beta-blocker therapy was introduced.

The clinical symptoms of palpitations and vertigo were observed in 50% of the study subjects with newly diagnosed AF. The remaining patients reported no complaints; these were also patients with silent atrial fibrillation (SAF).

Episodes of SAF were detected between day 1 and day 25 after starting ECG monitoring.

In the majority of patients (approx. 85%) with newly diagnosed AF (five of six study subjects), supraventricular extrasystoles were present in the 48 h Holter recording performed during stroke-related hospitalization.

Out of the analyzed parameters that increase the probability of, A.F.; only supraventricular extrasystoles proved to be an independent factor regarding an increased risk of AF [OR 1.046, CI 95% 1.016–1.071, *p*-value < 0.01].

A model, which increases the risk of AF detection while considering coexisting parameters without the dominance of a single parameter, was developed (Table 3).

**Figure 1:**
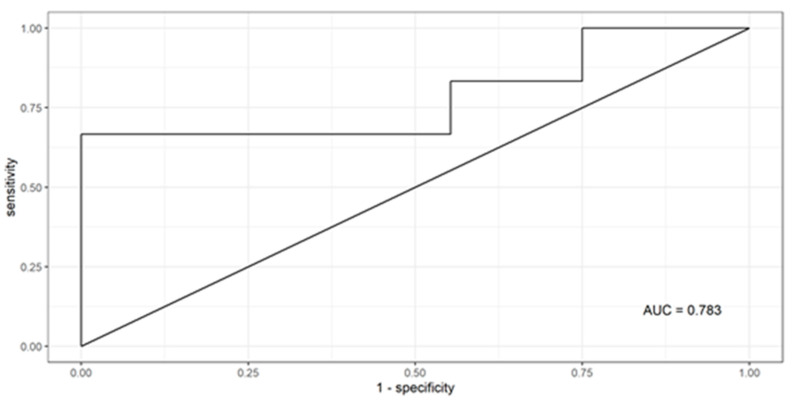
ROC curve of the binary logistic regression model for prediction risk of atrial fibrillation.

Sociodemographic characteristics (i.e., age) and the patient’s clinical profile (i.e., coronary artery disease) increase the probability of AF detection. For these parameters, the good predictive quality of AF was obtained (AUC = 0.783), (Figure 1).

## 4. Discussion

The most significant result of this study is the finding that among patients with ESUS, identified through an extended diagnosis of stroke cause, the incidence of AF is 4%. The presence of atrial supraventricular extrasystoles in the 48 h Holter recording performed within three months from stroke onset proved to be the only independent parameter to increase the probability of an AF diagnosis. The likelihood of AF detection was also in-creased by compiling the sociodemographic and clinical features of ESUS patients, including artery disease (coronary or peripheral), arterial hypertension, diabetes mellitus and the location of the stroke in the anterior cerebral circulation. In our study, AF was found only in patients with ESUS in the course of LVO.

AF is the most common cardiac arrhythmia characterized by an increased risk of thromboembolic complications, stroke included.

About 15% of stroke patients are diagnosed with AF before experiencing acute cerebral ischemia [2,3]. The use of an ECG and/or ECG Holter monitor as part of diagnostic procedures during hospitalization due to stroke reveals de novo AF in 5% of patients (3.8–6.5%) and, on average, in an additional 11% of patients (5.6–17.2%) undergoing diagnostic procedures after hospitalization [4].

As the prevalence of AF is estimated to increase two-to-three -fold due to increasing life expectancy in the general population, it is advisable to proactively identify patients with undiagnosed AF [18,19]. Patients with embolic stroke, i.e., ESUS, require particular attention. Approximately 16% of cryptogenic stroke cases are still related to AF because AF remains undiagnosed [20]. According to recent reports, apart from non-stenotic supracardiac atherosclerosis and neoplastic disease, AF is the leading cause of ESUS. Since the formulation of the ESUS concept in 2014, views regarding the prevalence of AF among ESUS patients and the efficacy of anticoagulation in unselected ESUS patients have been revised. Although the prevalence of AF in this group of patients is lower than originally expected, there is still an urgent need to look for arrhythmias, considering the risk of re-current stroke and the readily available options for effective patient management in secondary stroke prevention.

However, we do not have an established diagnostic formula for AF in patients with cryptogenic stroke. The European Society of Cardiology (ESC) recommends 24 h Holter monitoring in patients with cryptogenic stroke and at least 72 h monitoring if the former fails to reveal AF [21]. The European Stroke Organisation (ESO) indicates the need for ≤30-day ECG monitoring in patients after ESUS [22]. According to the American Stroke Association (AHA), when searching for signs of, A.F.; cardiac rhythm monitoring should be started on the first day of stroke [23]. The closer an episode of AF occurs to the index stroke or the longer an episode lasts, the more likely it is that they are causally associated. Episodes that are chronologically distant from the stroke or one of shorter duration are more likely to have no etiological association [24]. For this reason, experts propose a refinement of the diagnostic assessment for ESUS to include automated cardiac rhythm monitoring in ESUS patients in the stroke unit.

In the presented study, AF was most often found within the first days of signal re-cording in patients included in the study within one month after stroke onset. In accordance with the observations of many authors, the prevalence of newly diagnosed AF is lower if the interval between stroke and the initiation of recording is longer (in 23% of patients, with recording on day 21 day after stroke; in 17% of patients, with recording on day 33; in 16% of patients, with recording on day 77) [24,25,26]. It is worth noting that AF episodes occurring shortly after stroke (within three days) may happen as a consequence rather than because of acute cerebral ischemia, which probably does not alter the risk of recurrent stroke.

Randomized trials to compare various diagnostic models are needed. As the incidents of paroxysmal AF can be extremely brief, continuous monitoring is probably of a greater diagnostic value than intermittent monitoring [27,28]. In the latter case, various repeat registration formulas are used to increase the rate of AF detection (most often in the first year after ESUS). According to the results of one meta-analysis (8715 post-stroke patients), de novo AF was diagnosed in 5% of patients with ECG recording time < 72 h; however, it was detected in 15% of patients when the recording was extended to seven days [29,30]. In our study involving a homogeneous group of patients monitored for 30 days, AF was detected in 4% of subjects. Such a relatively low percentage may result from the fact that qualified patients had undergone extensive diagnostic procedures for AF and that patients after TIA were not eligible for the study. Additionally, the mean age of the study was below 55.

To date, observational studies using ECG monitoring in an outpatient setting, which differed in terms of the time of monitoring initiation after stroke, the duration of such monitoring and the methods applied, have demonstrated the rate of AF detection in patients with cryptogenic stroke or TIA at 17% (range 9–25%), depending on the type of study population [31]. Similarly, patients with AF make up one quarter of the total population of ischemic stroke patients.

In our study, supraventricular extrasystoles in ECG proved to be the only independent factor for AF detection. The association between premature atrial complexes in ECG and AF detection in the course of further diagnostic procedures in ESUS patients has also been shown by other authors [32,33,34,35,36].

In the presented study, we also found that several concomitant parameters formed a factor for increasing the rate of AF detection. Identifying specific patients with ESUS and AF is difficult. In our study, older age, the burden of vascular and metabolic comorbidities, as well as the stroke location in the anterior cerebral circulation, were associated with a higher likelihood of post-stroke AF. The above results are consistent with those obtained by other authors [37,38]. Although numerous clinical prediction scores based on such factors have been proposed, only a moderate predictive ability has been achieved at an individual level [39]. The use of clinical factors alone is unlikely to be sufficient for identifying the AF-ESUS subgroup of patients [39]. The analysis of the NAVIGATE study does not indicate any interaction between anticoagulant therapy and HAVOC score, which is intended to predict the risk of atrial fibrillation after stroke [40].

Two large international randomized clinical trials, NAVIGATE ESUS and RESPECT ESUS, failed to show any benefit with direct oral anticoagulant therapy over aspirin in preventing recurrent ischemic stroke [8,9]. However, it is still possible that patients with ESUS and a cardiogenic thromboembolic risk profile may benefit from anticoagulants. Our study revealed the benefits from anticoagulation even in AF patients with a high risk of intracranial bleeding [41]. The ESUS criteria probably need modifications. Anticoagulation therapy has been shown to reduce stroke due to device-detected subclinical AF. However, the number of patients with subclinical AF consists of individual patients with a different spectrum of baseline risk. We need to identify the risk factors and patient clinical profiles that will help distinguish patients who are at high risk of stroke and predict the benefits of using oral anticoagulation. According to the results of NOAH-AFNET 6 and ARTESiA trials, using anticoagulant therapy in subclinical AF patients is associated with a reduction in ischemic stroke of about 32% but with an increase in major bleeding of about 62% [42,43,44,45].

The use of serum cardiac biomarkers is a more promising strategy for identifying a subgroup of patients with ESUS who are at a high risk of future AF-related stroke and who might benefit from anticoagulant therapy. Although the results obtained by researchers differ slightly, troponin, I.; B-type natriuretic peptide (BNP) and N-terminal pro-BNP (NT-proBNP) have been seen in several studies as biomarkers of subsequent AF [46,47,48,49]. Midregional Proatrial Natriuretic Peptide (MRproANP) is a new and promising biomarker [50,51].

The AF-ESUS score, which was developed specifically for the prediction of AF in patients with ESUS, combines several of the echocardiographic biomarkers. It has a high sensitivity and a high negative predictive value in the identification of patients with ESUS at a low risk of new incident AF. Patients scoring ≥ 1 point were shown to be good candidates for prolonged cardiac rhythm monitoring [52].

Identifying the parameters that increase the risk of AF listed above does not require the use of diagnostic methods other than those routinely applied during the hospitalization of ESUS patients.

Physicians engaged in the diagnosis and therapy of ESUS patients should be alert to the parameters that increase the likelihood of AF occurrence.

When evaluating the clinical profiles of patients and their ECG results, we can identify a group of arrhythmia-prone patients and have them undergo prolonged cardiac rhythm monitoring.

The value of our study is clear from the fact that AF prevalence and AF predictors were found in subjects selected from a group of patients with cryptogenic stroke as defined by ESUS. Our findings are consistent with those of other authors, and we confirm that the telemetry is possible for cardiac monitoring [53].

Limitations of the study: No analysis of echocardiographic parameters (including the left atrium dimension) was conducted, as the number of procedures available was limited. The telemetric system used was limited regarding obtaining detailed analysis of heart rate variability.

## 5. Conclusions

The use of telemetry heart rhythm monitoring in an outpatient setting can detect AF in 4% of ESUS patients who have undergone prior diagnostic procedures for cardiogenic embolism.

Supraventricular extrasystoles significantly increases the likelihood of AF detection in patients with ESUS within three months following stroke.

Comorbid coronary artery disease, diabetes and hypertension, rather than a single-factor clinical burden, increase the likelihood of AF detection in older ESUS patients.

ESUS in the course of large vessel occlusion is probably associated with an increased likelihood of cardiogenic embolism.

## Figures and Tables

**Table 1:** The characteristic of study group.

Parameter	Value
Mean age, med. [ref.]	52.71 ± 7.29, 54 [18–81]
F, n (%)	58 (40.27)
Coronary artery disease, n (%)	19 (13.19)
Diabetes mellitus, n (%)	27 (18.75)
NIHSS on day 1 of stroke, med. [ref.]	14 [6–24]
NIHSS during study enrolment, med. [ref.]	7 [6–18]
mRS during study enrolment, med. [ref.]	2.5 [0–4]
Arterial hypertension, n (%)	88 (61.51)
Lipid disorders, n (%)	37 (25.69)
Past myocardial infarct, n (%)	3 (2.08)
Peripheral artery disease, n (%)	4 (2.76)
Nicotinism, n (%)	35 (24.14)
Stroke-onset recruitment time (day), mean, med. [ref.]	37.2 ± 7.4, 41 [9–90]
Abnormalities in ECG (%) *	
sinus bradycardia	33 (22.70)
supraventricular extrasystoles	44 (30.34)
increased maximum P-wave duration	10 (6.89)
prolonged PR interval	41 (28.27)
Reperfusion therapy, n (%)	
rtPA	44 (30.55)
MT	19 (13.19)
rtPA+ MT	20 (13.88)
Location of ischemic lesion, n (%)	
Left hemisphere	92 (63.88)
Right hemisphere	11 (7.63)
Multifocal	22 (15.27)
Brainstem	19 (13.19)
OCST classification, n (%)	
TACI	29 (20.13)
PACI	81 (56.25)
POCI	34 (23.61)
Secondary stroke prevention, n (%)	
aspirin	89 (61.8)
clopidogrel	27 (18.75)
aspirin + clopidogrel	17 (11.8)
aspirin + cilostazol	2 (1.38)
aspirin + ticagrelor	5 (3.47)
aspirin + rivaroxaban	4 (2.78)

* performed during stroke-related hospitalization. ECG—electrocardiogram, mRS—modified Rankin Scale, MT—mechanical thrombectomy, NIHSS—National Institutes of Health Stroke Scale, OCST—Oxfordshire Community Stroke Project, PACI—partial anterior circulation infarct, POCI—posterior circulation infarct, rtPA—reversible tissue plasminogen activator, and TACI—total anterior circulation infarct.

**Table 2:** Parameters of telemetry heart rate monitoring.

Parameter	Value
Minimum HR/min., mean, med. [ref.]	51 ± 14, 50 [33–73]
Maximum HR/min., mean, med. [ref.]	126 ± 6, 126 [82–159]
Mean HR/min., mean, med. [ref.]	70 ± 13, 70 [50–87]
Time from device fitting to AF detection (days), mean, med. [ref.]	10.67 ± 1.0, 8 [1–25]
Duration of first AF episode (min.), mean, med. [ref.]	446.50 ± 1.0, 330.50 [35–1440]
Other cardiac arrhythmias, n (%)	
supraventricular extrasystoles	59 (40.69)
ventricular extrasystoles (ventricular bigeminy, non-sustained ventricular tachycardia)	25 (17.24)
Symptoms pointing to cardiac arrhythmias, n (%)	
heart palpitations	19 (13.10)
vertigo	8 (5.52)

**Table 3:** Sociodemographic and clinical parameters that increase the risk of AF diagnosis in ESUS patients.

Coefficient.	OR	CI 95%	*p*-Value
Sex	1.095	(0.17–9.33)	0.925
Age	1.097	(1–1.23)	0.079
Coronary artery disease	0.097	(0.007–2.28)	0.076
Arterial hypertension	2.619	(0.30–65.63)	0.445
Diabetes mellitus	0.515	(0.01–4.83)	0.616
Peripheral artery disease	1.321	(0.17–28.29)	0.814
Stroke in anterior cerebral circulation	0.801	(0.37–1.20)	0.452

## Data Availability

Data are contained within the article.
